# Pressure-relief joints of initial support structural system used in tunnels with high-stress surrounding rock

**DOI:** 10.1371/journal.pone.0297668

**Published:** 2024-04-04

**Authors:** Zhiming Xu, Yashuang Bai

**Affiliations:** Institute of Civil Engineering, Southwest Forestry University, Kunming, China; Jamia Millia Islamia, INDIA

## Abstract

To address the problem of large deformations in weak surrounding rock tunnels under high ground stress, which cause damage to initial support structures, this study proposes a novel type of circumferential pressure-relief joint based on the concept of relieving deformation pressure of the surrounding rock. Key parameters of the pressure-relief joint, such as initial bearing capacity peak, constant bearing capacity, and allowable pressure-relief displacement, were obtained through numerical simulations and laboratory experiments. A comparison was made between the mechanical characteristics of rigid joints and the new type of pressure-relief joint. The applicability of the pressure-relief joint was verified through field tests, monitoring the surrounding rock pressure, internal forces in the steel frames, and the convergence displacement of the support structure. The results show that: (1) In the elastic stage, the stiffness of the new pressure-relief joint is similar to that of rigid joints. In the plastic stage, rigid joints fail directly, whereas the pressure-relief joint can control deformation and effectively release the deformation pressure of the surrounding rock while providing a constant bearing capacity. (2) The right arch foot in the experiment had poor rock quality, leading to high stress in the steel frame and significant horizontal displacement. After the deformation of the pressure-relief joint, the stress in the surrounding rock and steel frame significantly reduced, and the rate of horizontal deformation of the support structure slowed down. (3) The vertical and horizontal final displacements of the pressure-relief joint in the experiment were 61mm and 15mm, respectively, which did not exceed the allowable deformation values. The components of the support structure remained intact, ensuring safety. However, this study has limitations: the design of the new pressure-relief joint only allows for a vertical deformation of 150mm and a horizontal deformation of 50mm, limiting the range of pressure-relief deformation.

## 1 Introduction

The soft surrounding rock with high in-situ stress is prone to extrusion and significant deformation. The extrusion of soft rock will cause the supporting structure to bear tremendous surrounding rock pressure, which often leads to concrete cracking, steel frame distortion, and other initial support failures [1,2]. Even major engineering disasters such as tunnel collapse occur. During the construction phase, such damage frequently occurs, such as the Jiazhuqing Tunnel of the Nankun Line in my country [3], the Muzhailing Tunnel in Gansu [4], the Tauern Tunnel in Austria [5], the Enasan Tunnel in Japan [6], the Gotthard Railway Tunnel [7], etc., severe extrusion and large deformation disasters occurred, resulting in slow construction progress [8].

In researching the destruction of initial support structures in soft rock tunnels under high ground stress and large deformations, methods commonly used include theoretical analysis [9,10], numerical simulations [11], and field experiments [12,13]. Numerical simulations often employ finite difference methods [14], finite element analysis [11], and boundary element methods [15–18]. Scholars both domestically and internationally have found through theoretical analysis, numerical simulations, and field tests that the occurrence of large deformations in weak surrounding rock under high ground stress is directly related to geological conditions, original rock stress, and tunnel support methods.

Due to the uncontrollable external factors such as geological conditions and original rock stress in actual engineering, in order to avoid the large extrusion deformation and damage of the tunnel, the bearing capacity of the supporting structure can only be improved. There are three existing tunnel support methods: timely rigid support, multi-layer rigid support and yield pressure support. Just-in-time rigid support refers to the timely use of large-rigidity support structures for support after tunnel excavation. Although this support method can achieve the purpose of suppressing the deformation of surrounding rock [6], the support structure is in a state of extremely high stress for a long time, and the rock The energy stored in the body cannot be released. When the energy accumulates to a certain level, the supporting structure will suffer brittle failure, such as the bolt being pulled off, the steel frame being twisted, or the concrete falling off or cracking [19–21]. The multi-layer rigid support is to erect the support structure in two or more layers. The rigidity of the first layer of the support structure is relatively small. When the deformation energy of the surrounding rock is released to a certain extent, the next layer of support is erected to strengthen the support. The overall stiffness of the structure makes the tunnel reach a stable state, but the rigid support is prone to brittle failure. After the first layer of the support structure is damaged, if the next layer of consent cannot be carried out in time, it will cause a tunnel collapse accident [22], the rigid support cannot make the soft rock extrusion large deformation tunnel reach a stable state [23].

The pressure-relief support allows the support structure to deform to release the surrounding rock pressure to ensure the surrounding rock’s stability. It is widely used in large-deformation tunnels extruded by soft rock. There are two primary structural forms of yielding support: a compressible layer is arranged between the surrounding rock and the lining, or a yielding member is placed on the circumference of the steel frame [24]. The first form of support is to absorb the energy generated by the surrounding rock’s deformation through the compressible layer’s deformation [25]. For example, Xiong-Yu Hu et al. [26] set up expansive soil between the surrounding rock and the lining to achieve pressure yielding. Foam concrete [27,28], ceramics [29], and other materials are used to make compressible layers, but the compressible layer is not clearly defined, and the construction process is complicated. The second form of support is to achieve pressure yielding by allowing the deformation of the pressure-relief member, such as slip [30] or pressure-relief [31–33]. For example, Zhang Chuanqing et al. [34], When the deformation is too large, the pressure rod is often pulled off. Dong Biao et al.[35]used U-shaped channel steel to make the pressure-relief member realize the sliding of the primary support member along the set path and reduce the stiffness of the direct support. However, the U-shaped pressure-relief member is complicated to process and has poor matching with the direct support structure of most tunnels. He Manchao [36] et al. designed a slip-type yielding pressure adaptive joint composed of high-strength preloaded bolts, an H-shaped steel frame, and channel steel. Under the action of complex stress, this joint is prone to deformation and failure of the rail joint. It loses its supporting capacity. Qiu Wenge et al. developed a resistance limiter, which uses the bending deformation of the vertical steel plate to release the surrounding rock pressure [37]. Still, the plastic deformation rate of the web of the resistance limiter is too fast, and the bearing capacity is unstable [38].

The above studies indicate that existing pressure-relief support structures still face challenges such as complex construction techniques, unstable pressure-relief deformation, and susceptibility of pressure-relief elements to failure. Therefore, the authors of this paper propose a support structure with new circumferential pressure-relief joints installed. The key performance parameters of these joints are explored through numerical simulations and laboratory experiments, comparing the data obtained through these two methods to validate the rationality of the pressure-relief joint design. In the high ground stress section of the Haba Snow Mountain Tunnel, three experimental arch frames were erected to conduct field experiments and analyze the on-site monitoring data, verifying the mechanical performance of the pressure-relief joints. This aims to provide reference and guidance for the design of initial support structures in soft rock tunnels under high ground stress.

## 2 Structure and design of pressure-relief joint

### 2.1 Joint construction

**Fig 1:**
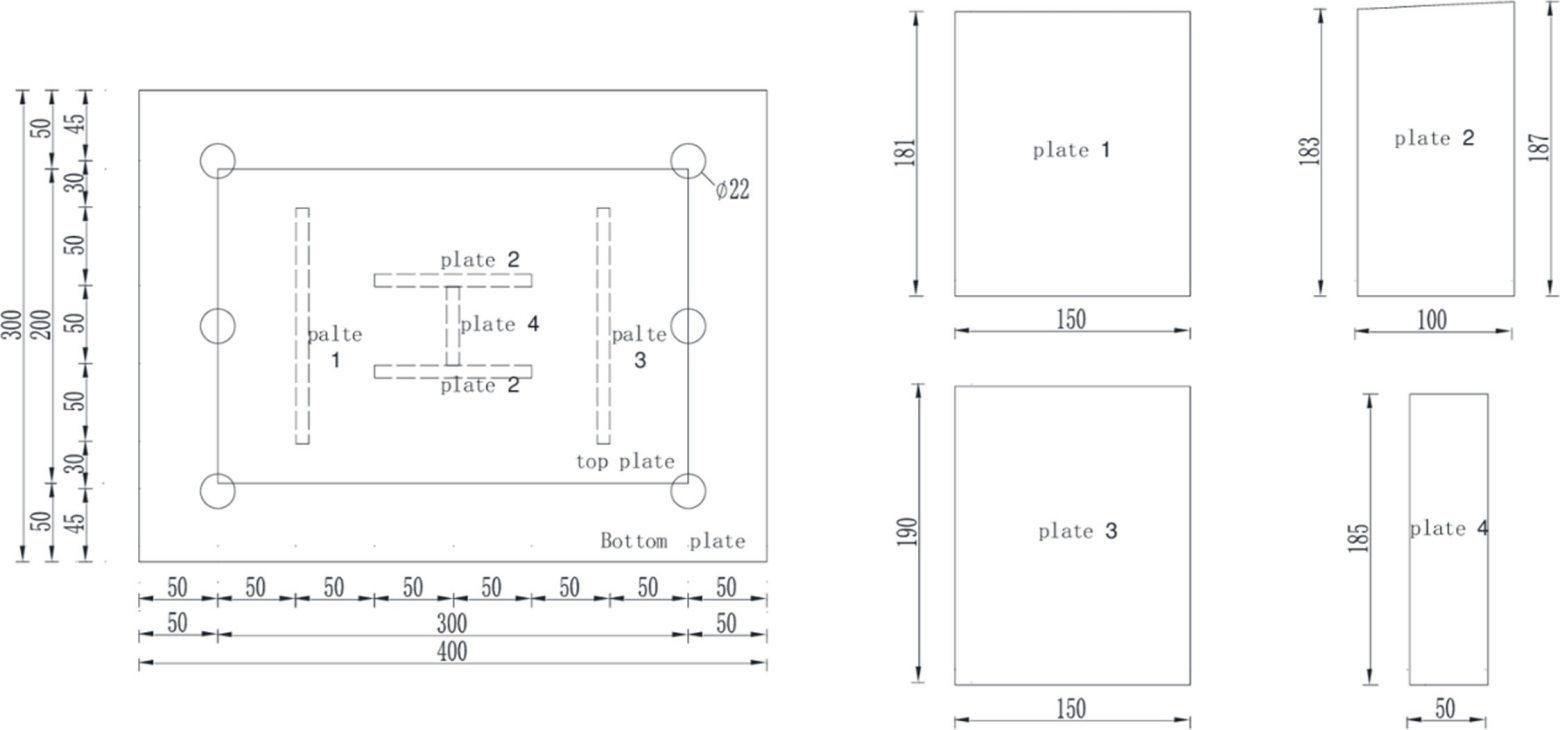
Schematic diagram of the structure of the pressure-relief joint.

**Fig 2:**
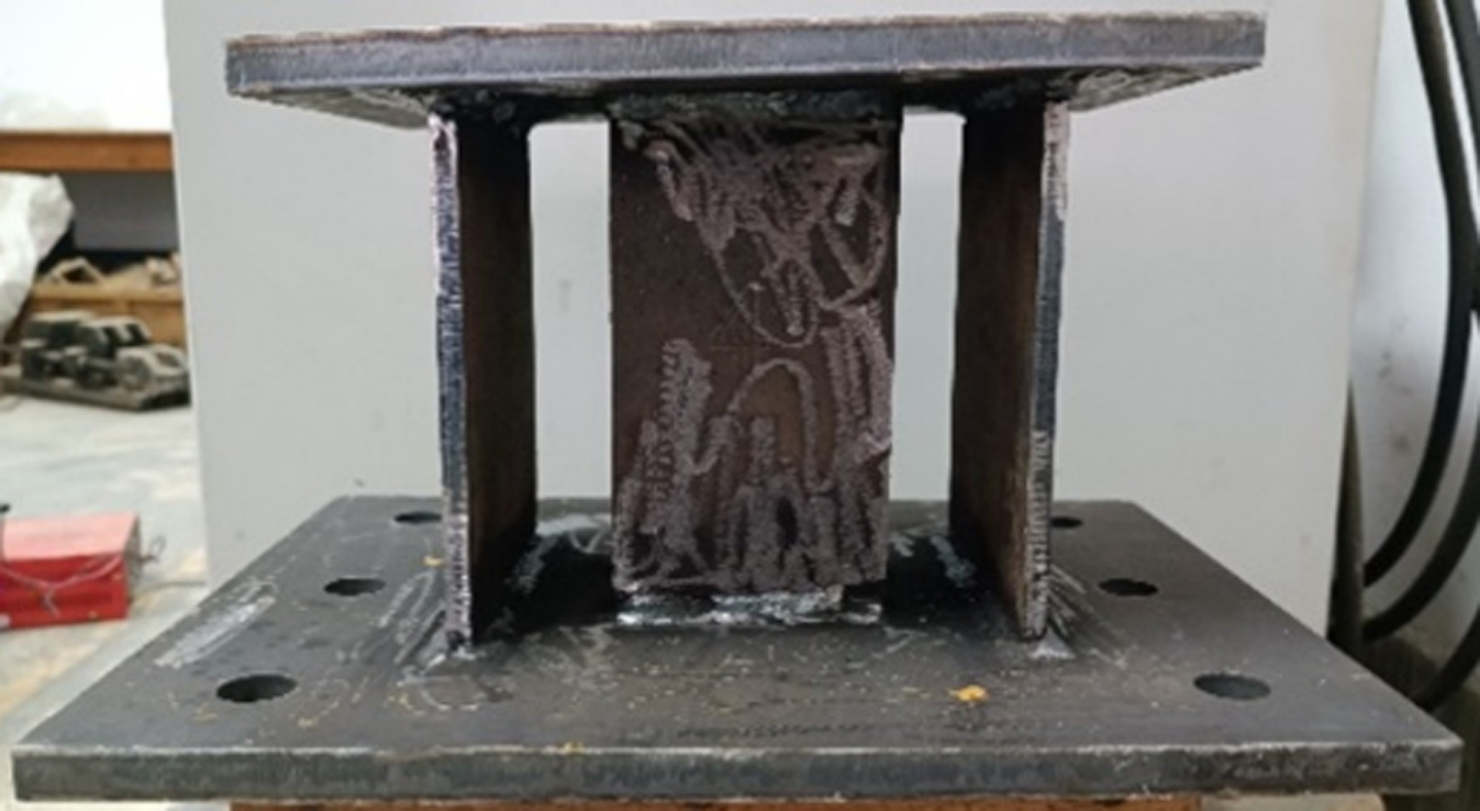
Pressure-relief joint sample map.

The new type of circumferential pressure-relief joint consists of three parts: the top plate, the bottom plate, and the web. The top and bottom plates are used as force transmission plates to transfer the load in the arch. The web is used as the stress plate, and the relief pressure is realized by plastic deformation during the stress process. Let the angle θ between the top plate and the bottom plate of the pressure-relief joint be 2.3° to simulate the curvature of the joint in the actual arch, and set 6 22mm bolt holes on the bottom plate of the pressure standard for connecting the steel frame, let the web and bottom plate of the pressure-relief joint, and the outer web (plate 1, plate 2, plate 3) and the top plate are connected by welding, the middle web (plate 4) and the top plate are planed tightly, and the schematic diagram of the pressure-relief joint structure is shown in Fig 1, and the joint is shown in Fig 2 Show.

### 2.2 Joint design principles

The design pressure-relief joint should meet the following requirements:

(i) The peak value of elastic bearing capacity perpendicular to the top plate and stiffness are slightly smaller than those of axial elastic bearing capacity of rigid joints. Assuming that the top plate of the pressure-relief joint is parallel to the bottom plate, the vertical bearing capacity is determined by the following formula,

$$F_{\text{vertical}}=\sigma A$$ (1)

In the formula: σ is the normal stress, A is the area where the normal stress acts.

Then, calculate the peak value *F*_total_ of the elastic bearing capacity of the pressure-relief joint with the curvature of the arch,

$$F_{\text{total}}=\frac{F_{\text{vertical}}}{cos\theta }$$ (2)

In the formula: *θ* is the angle between the top plate and the bottom plate.Lateral shear force *F*_horizantal_,

$$F_{\text{horizontal}}=F_{\text{vertical}}\times tan\theta$$ (3)

(ii) When selecting the web section size, let the shear bearing capacity and shear stiffness of the web of the pressure-relief joint be greater than the shear bearing capacity and shear stiffness of the connecting member.
(iii) The shear bearing capacity of the bolt is greater than the shear bearing capacity of the pressure-relief joint,Bolt Shear Capacity:

$$V_{2}=\text{0.9}n_{f}\mu P$$ (4)

In the formula: *n*_*f*_ is the number of force-transmitting friction surfaces, taking *n*_*f*_ = 2, μ is the anti-slip coefficient, taking μ = 0.30, P is the design value of high-strength bolt preload, taking P = 190KN.

The pressure-relief joint shear capacity:

$$V_{3}=\frac{[\tau ]It}{S^{*}}$$ (5)

In the formula: [τ] is allowable shear stress, t is the thickness of the web, I is the moment of inertia, S* is the static moment, take $[\tau ]=\frac{\text{0.58}f_{y}}{\text{1.1}}$, and *f*_*y*_ is the yield strength of the steel.

(iv) For the example in this paper, *F*_total_ = 704.6KN, *F*_horizontal_ = 28.3KN. In subsequent calculations, the *F*_horizontal_ is the shear force *V*_1_ acting on the pressure-relief joint. The shear bearing capacity of a single bolt obtained from formula (4) is 102.67KN, and the shear bearing capacity of the pressure-relief joint obtained from formula (5) is 90KN.

$$V_{1}<V_{3}<V_{2}$$

The hoop stress acting on the pressure-relief joint will not cause the shear failure of the web and the shear failure of the web before the bolt.

## 3 Pressure-relief joint numerical simulation

**Fig 3:**
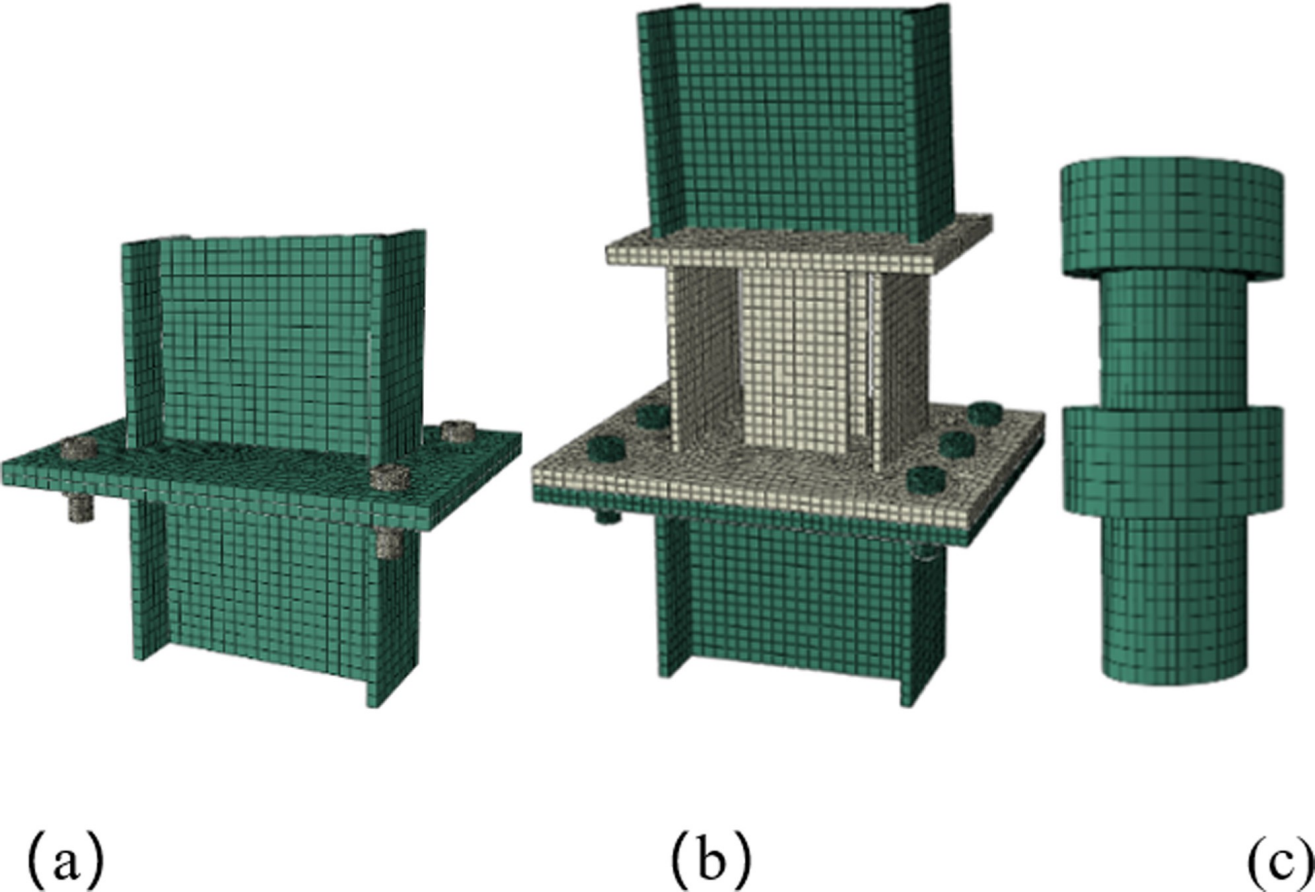
Finite element analysis model. (a) Rigid joint, (b) pressure-relief joint, (c)bolt.

The ABAQUS software was used to establish analytical models for both rigid and pressure-relief joints, with components divided using C3D8R elements. The mesh size of the bolts was set to approximately 2mm, while other components were set to about 8mm. Details of the analysis models and their mesh divisions are shown in Fig 3.

### 3.1 Model overview

The rigid joint analysis model assumes that bolts and flange plates connect two sections of I-25b steel, and an inclination angle is set on the upper part of the rigid joint to ensure the same direction of force as the pressure-relief joint. The steel constitutive model is ideal elastic-plastic, C3D4 is used to mesh the bolts, and C3D8R is used for meshing the rest of the components. All components are Q235-B Steel.

The analytical model of the pressure-relief joint comprises two parts, the I-25b steel, and the pressure-relief joint when simulating the natural stress situation of the pressure-relief joint in the tunnel. The top and bottom plates of the pressure-relief joint are made of Q235-B steel, and the web is made of LY160 steel. The web and bottom plate, as well as the outer web and the top plate, are connected by penalty, and the middle web and the top plate are in hard contact. The constitutive model and mesh were used. The type is the same as that of the rigid joint model.

### 3.2 Vertical loading simulation

**Fig 4:**
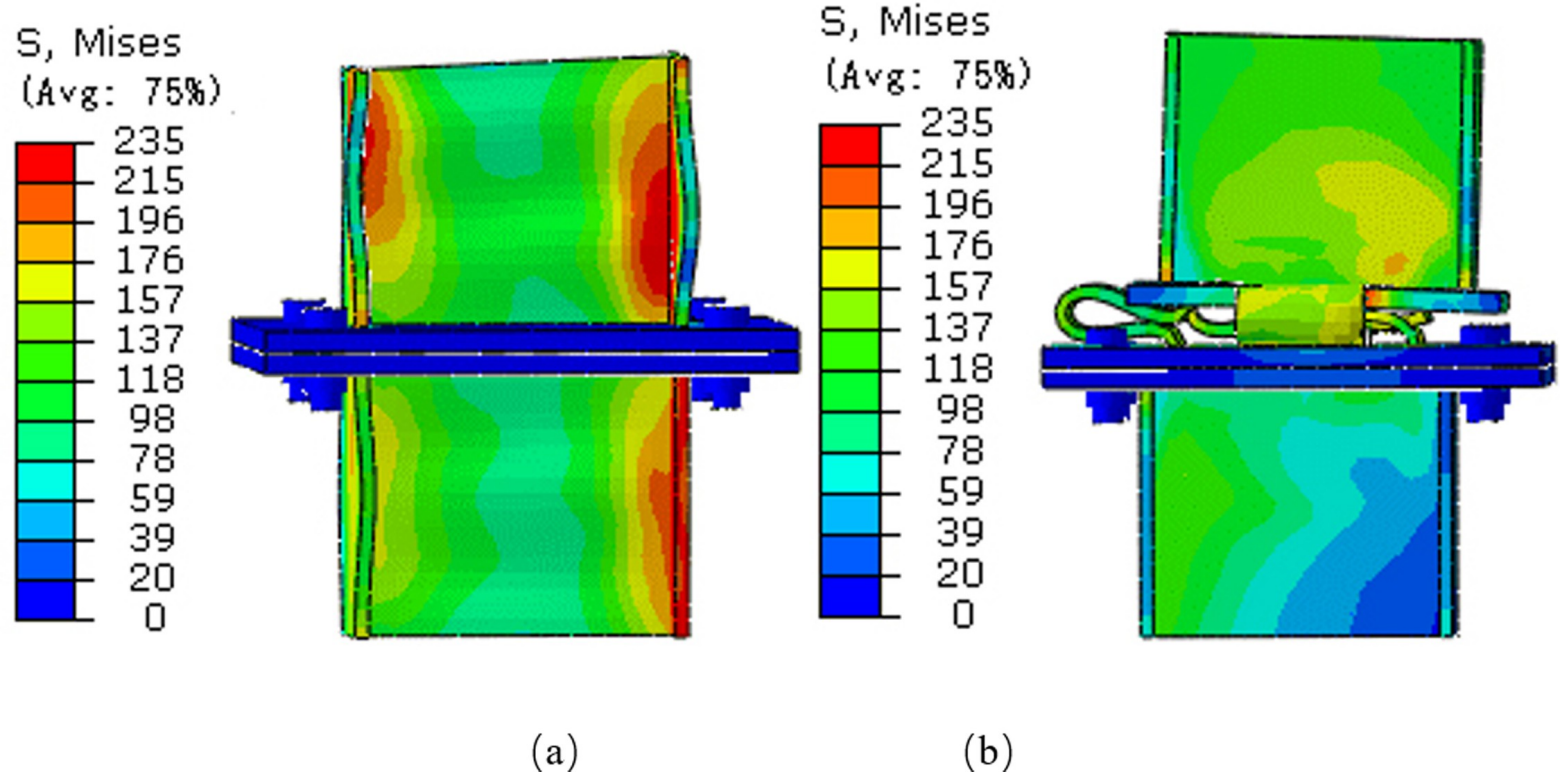
Mises stress nephogram of two kinds of joints (Unit: MPa). (a) Rigid joint, (b) Pressure-relief joint.

The displacement control method is used for vertical loading. An axial displacement of 10 mm is applied to the section of the rigid joint, and an axial displacement of 160 mm is applied to the top plate of the pressure-relief joint to obtain the Mises stress cloud diagram (Fig 4). It can be seen from Fig 4. that under this load, both the rigid joint and the pressure-relief joint have undergone plastic deformation, and the pressure-relief joint is compacted.

### 3.3 Joint comparison

**Fig 5:**
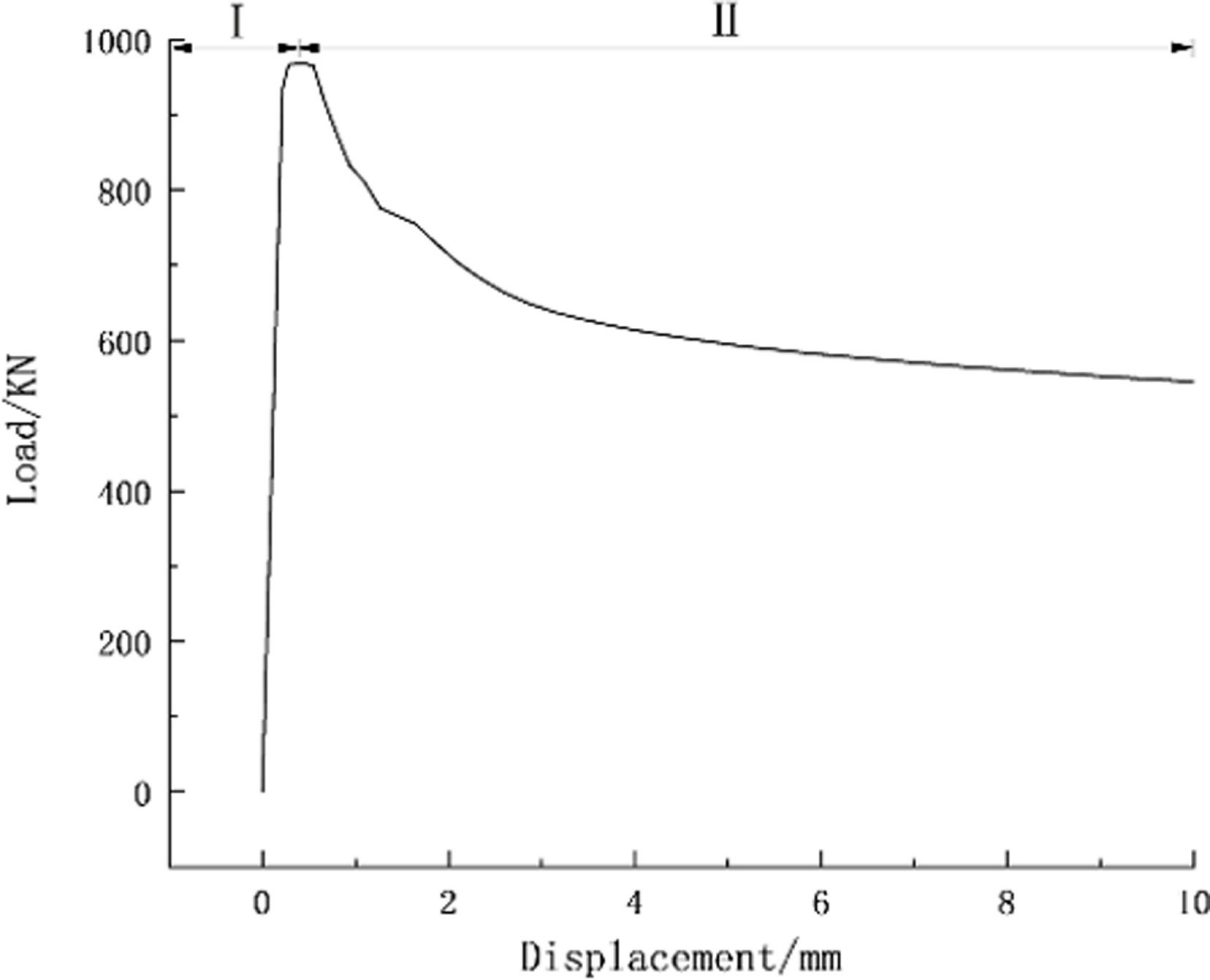
Numerical simulation of load-displacement curves for rigid joints.

**Fig 6:**
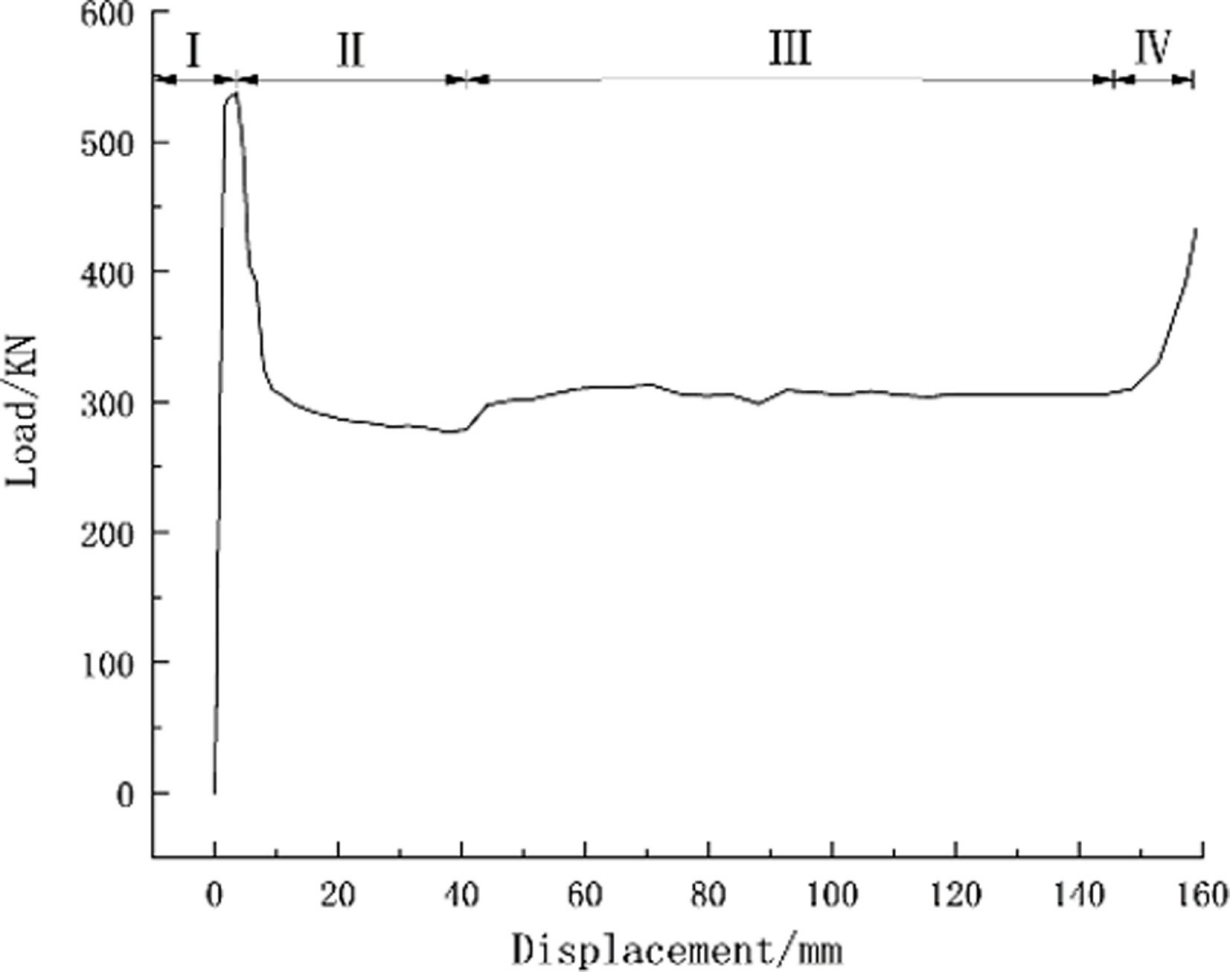
Numerical simulation load-displacement curve of pressure-relief joint.

The load-displacement curves for the rigid and pressure-relief joint are plotted (Figs 5 and 6). The deformation process of rigid joints divides into two stages in Fig 5. The bearing capacity of rigid joints increases linearly with displacement in the stage of rising bearing capacity (I). The bearing capacity of rigid joints decreases rapidly during the stage of falling bearing capacity as the plastic deformation of rigid joints increases (II).

In Fig 6, the deformation process of the pressure-relief joint is divided into four stages: in the elastic deformation stage, the bearing capacity of the pressure-relief joint increases with the increase of displacement (I), in the plastic deformation stage, the bearing capacity increases with the plastic deformation of the pressure-relief joint. While decreasing (II), in the constant bearing capacity stage, the bearing capacity is stable with the plastic deformation of the web (III), in the compaction closing stage, the pressure-relief joints are compacted (IV).

It can be seen from the numerical simulation results that the critical load value when the rigid joints appear with plastic deformation is 968KN and the critical load value when the pressure-relief joints appear under plastic deformation is 537KN. When the rigid joint enters the plastic state, it means that under the action of the surrounding rock pressure, the rigid support will intrude at any time, when the pressure-relief joint enters the plastic state, the bearing capacity remains stable after the compressive deformation is released. There is a slight increase in the bearing capacity of the plate during compacting.

## 4 Pressure-relief joint indoor test

### 4.1 Test overview

A hydraulic universal testing machine was used for the loading test, and the test conditions were the same as the numerical simulation conditions. The maximum loading force of the testing machine is 1000KN. The loading mode is program control, the loading load is 700kN, and the speed is 1KN/s.

### 4.2 Test results

**Fig 7:**
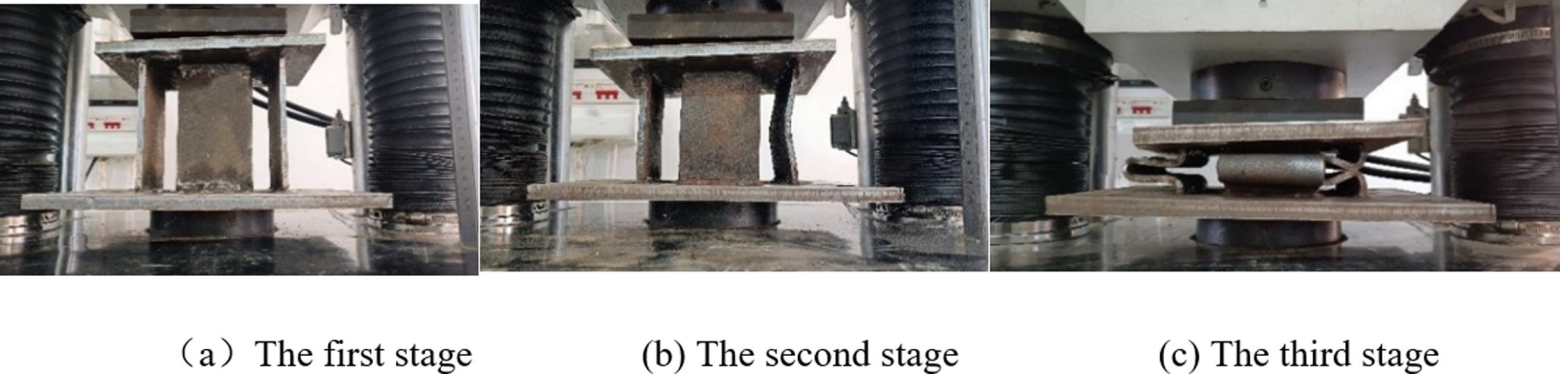
Pressure-relief joint deform. (a) The first stage, (b) The second stage, (c) The third stage.

The deformation process of the pressure-relief joint web is divided into three stages, as shown in Fig 7. In the first stage, the web does not deform, and the joints reach the peak initial bearing capacity, in the second stage, the web is bent and deformed, and the bearing capacity decreases. The joints can provide the constant bearing capacity, in the third stage, the webs are superimposed on each other so that the pressure-relief joints are compacted and can continue to bear.

### 4.3 Comparison of results

**Fig 8:**
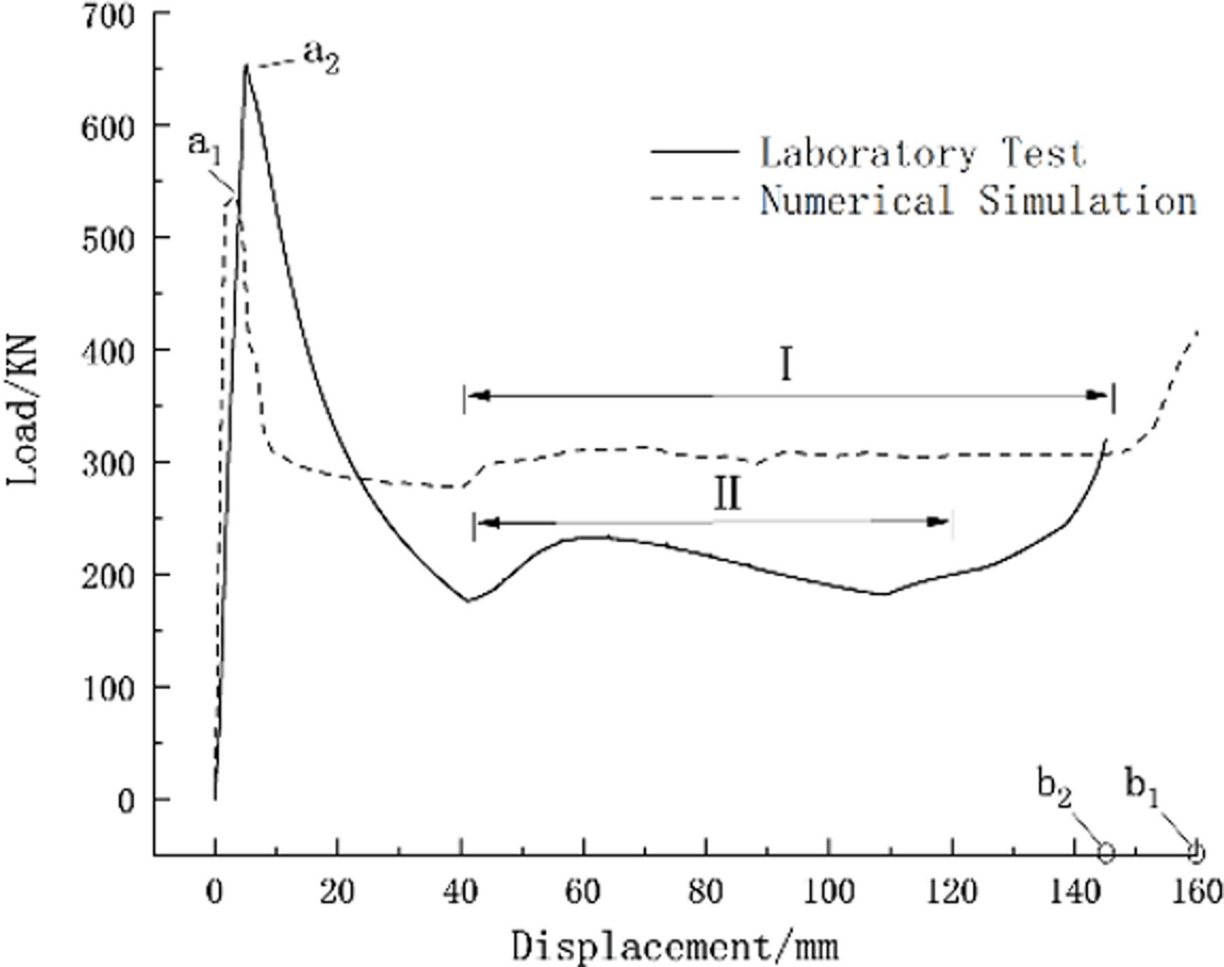
Comparison of load-displacement curves for active deformations.

The load-displacement curves of the pressure-relief joint experiments were drawn and compared with numerical simulation load-displacement curves (Fig 8). It was found that the initial bearing capacity peak of the pressure-relief joint obtained by numerical simulation was about 537KN (at point a_1_), the constant bearing capacity was about 306KN (Phase I), and the allowable pressure-relief displacement was about 160mm (at point b_1_). In contrast, the laboratory experiment yielded an initial bearing capacity peak of about 654KN (at point a_2_), a constant bearing capacity of about 220KN (Phase II), and an allowable pressure-relief displacement of about 145mm (at point b_2_).

The numerical simulation results and experimental results of the pressure-relief joints show high consistency, yet there are still some numerical differences. The reasons for these differences include residual stress from welding in the pressure-relief joints and the fact that during the loading process of the testing machine, the pressing plate first contacts the higher part of the pressure-relief joint’s top plate (due to the design incline of the top plate). The maximum axial load obtained in the numerical simulation was 83% of the experimental result, while the maximum vertical deformation was 1.1 times that of the experimental result.

## 5 Field trial program

### 5.1 Project overview

The pressure-relief joints designed in this paper are used in the Haba Snow Mountain Tunnel. The tunnel has a total length of 9523m and a maximum buried depth of 1155m. The starting and ending mileage is DK52+183-DK61+706, of which the DK59+140~+300 section. The stratum lithology is Gray-green schistose basalt, flaky structure, locally layered and interbedded in lamellae. The rock mass undergoes schistose metamorphism, and the thickness of the lamellae is 0.05–0.2m. The rock mass is relatively fragmented to broken, and joints are developed. The rock mass is fragmentary mainly and breccia with poor integrity. The groundwater is developed, the stability of the surrounding rock is poor, and the grade of the surrounding rock is comprehensively judged to be grade V.

**Fig 9:**
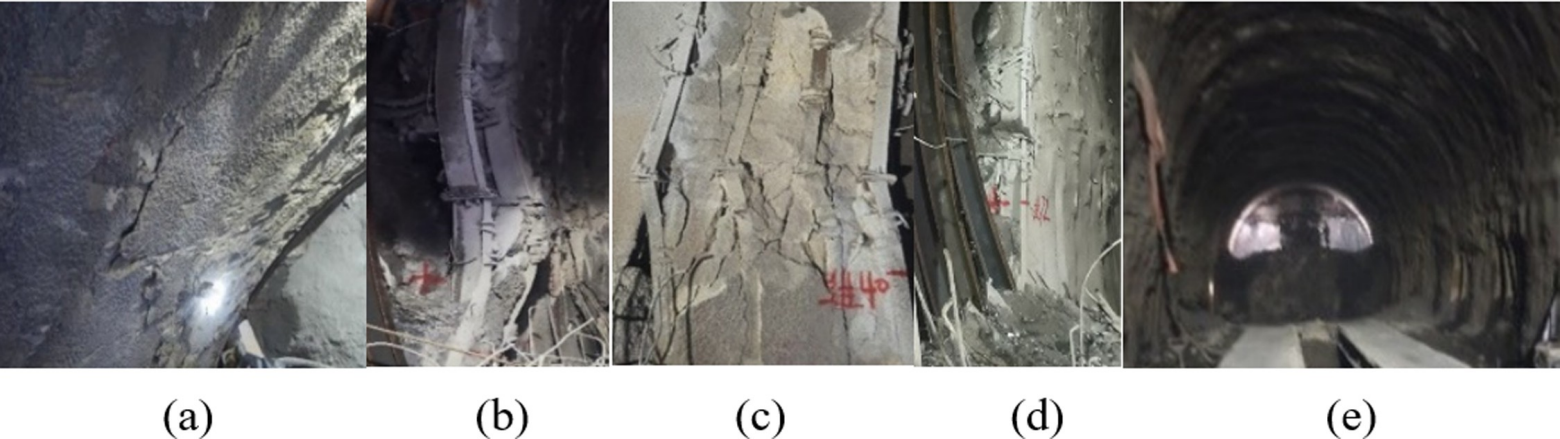
Destruction diagram of the original initial support structure. (a)Cracked concrete, (b)Steel frame is crushed, (c)Invade limit of the left steel frame, (d)Right steel frame intrusion limit, (e)Damage to the overall structure.

Section DK59+140~+300 of Haba Snow Mountain Tunnel was adjusted from pre-designed V-grade A-type composite lining to large-deformation ⅢB-type composite lining. The inner and outer layers were equipped with 25b-shaped steel frames to strengthen support, with a spacing of 0.6m/tree. The arch is provided with Φ42 small conduits for advanced support, with a circumferential spacing of 0.4m and a longitudinal spacing of 1.8m, each with a length of 3.5m and 25 per ring. When the steps were constructed to DK59+228, the initial support structure of the DK59+252~+300 section was damaged. The damage was manifested as concrete cracking from the vault to the left and right arch waists. The steel frames at the left and right arch feet, containing the left and the right side walls, were all damaged. It was crushed and invaded the boundary, and the original initial support structure was damaged, as shown in Fig 9.

### 5.2 Joint installation and test monitoring

**Fig 10:**
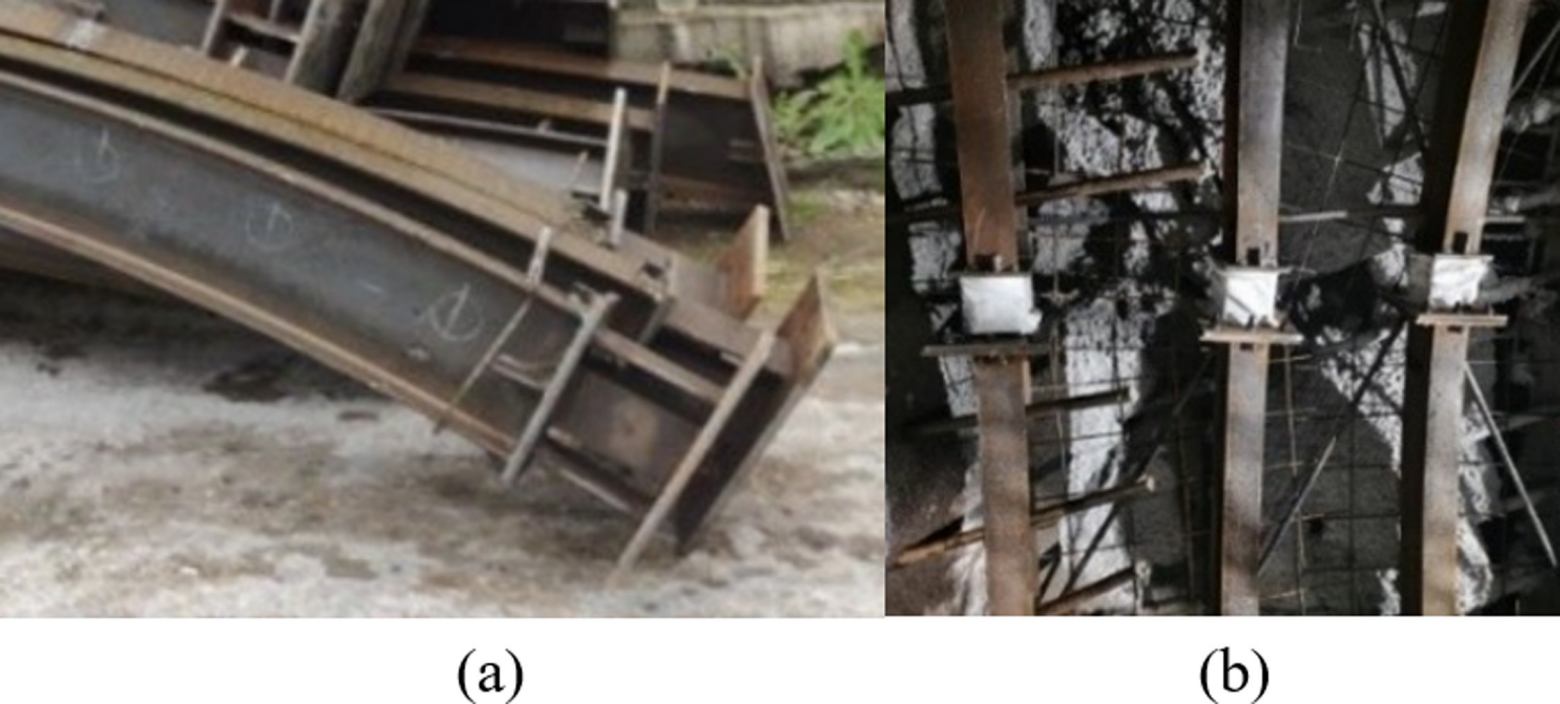
Pressure-relief joint install. (a) Test steel frame vault unit, (b) Connection diagram of joints and vaults and vaults.

DK59+220.8~+222 is selected as the test section. The test plan is: to install one pressure-relief joint at the connection between the dome and the left and right arch waist of the 25b steel frame, and the other processes are still constructed according to the original design. So that the pressure-relief joint is installed as shown in Fig 10.

**Fig 11:**
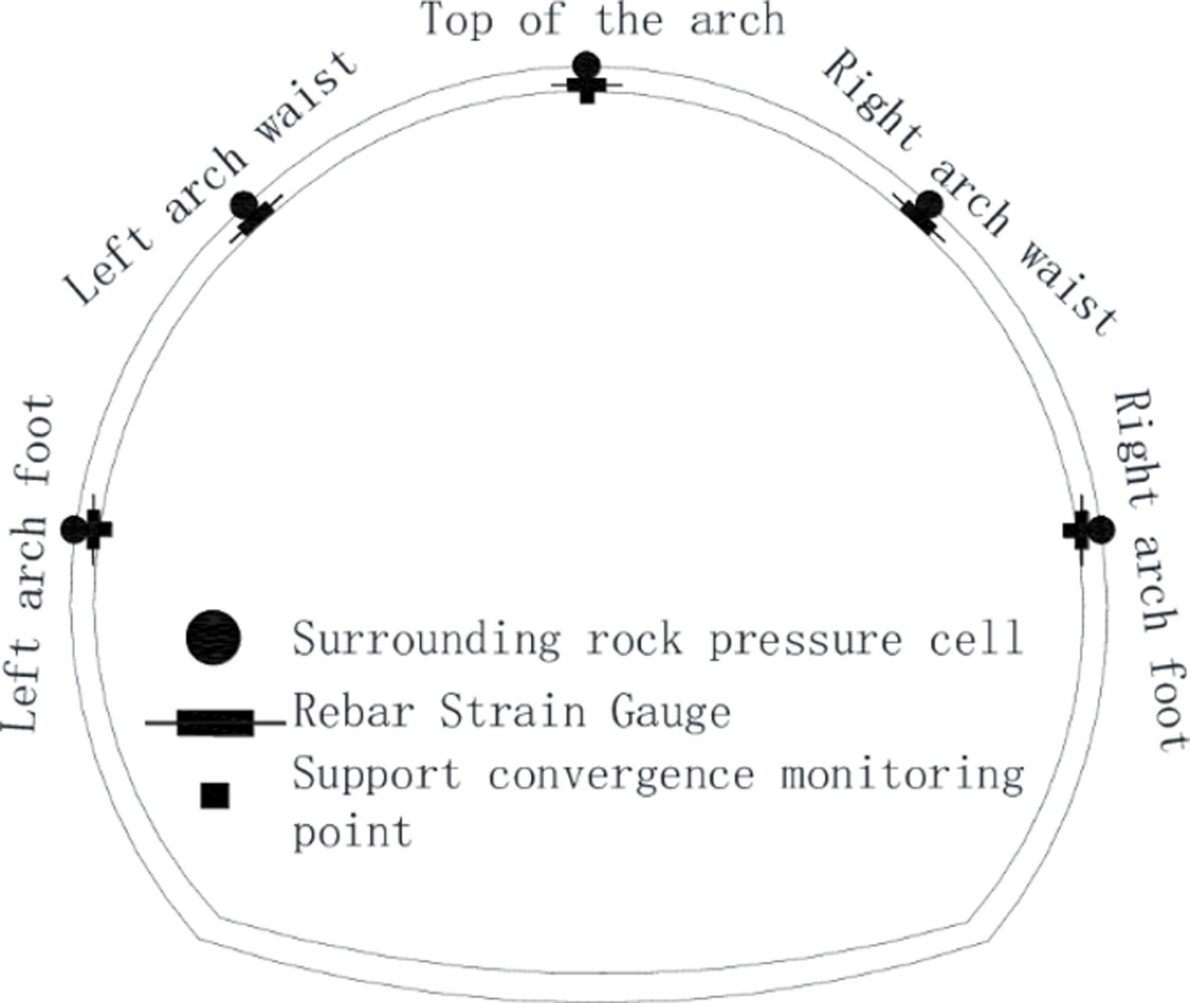
Schematic diagram of the installation position of monitoring components.

The surrounding rock pressure cells and steel strain gauges are used to monitor the contact pressure between the surrounding rock and the initial support, the internal force of the initial support steel frame, and the total station to monitor the support convergence. There are three monitoring sections in the test section. The monitoring positions of the surrounding rock pressure and the internal force of the steel frame are the vault, the left, and right arch waists, and the left and right arch feet. The support convergence monitoring positions are the vault (vault settlement) and the left and right arch feet (peripheral horizontal convergence)—the installation positions of monitoring components, such as Fig 11.

### 5.3 Monitoring data

**Fig 12:**
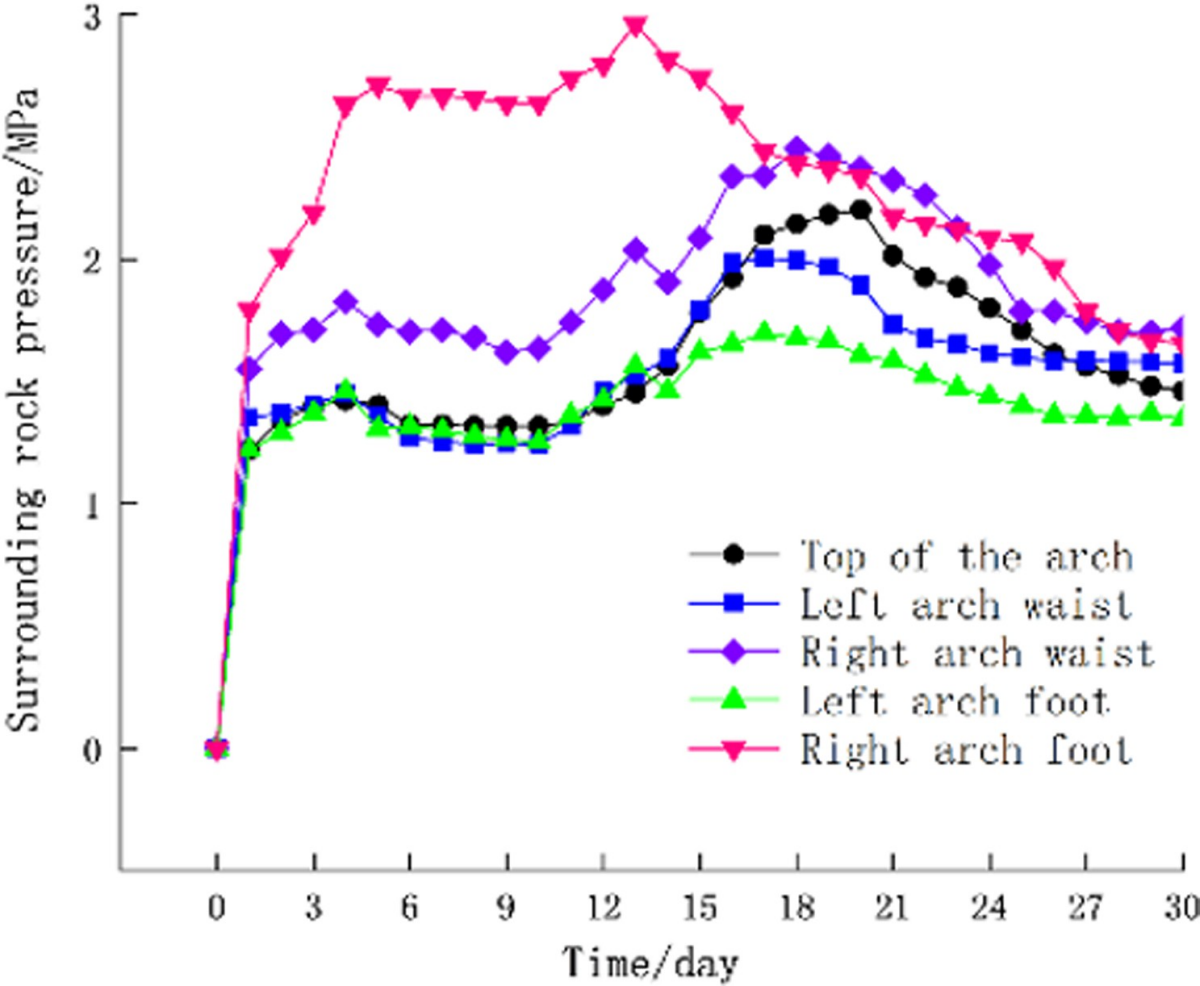
Time-history curve of surrounding rock pressure at monitoring section.

**Fig 13:**
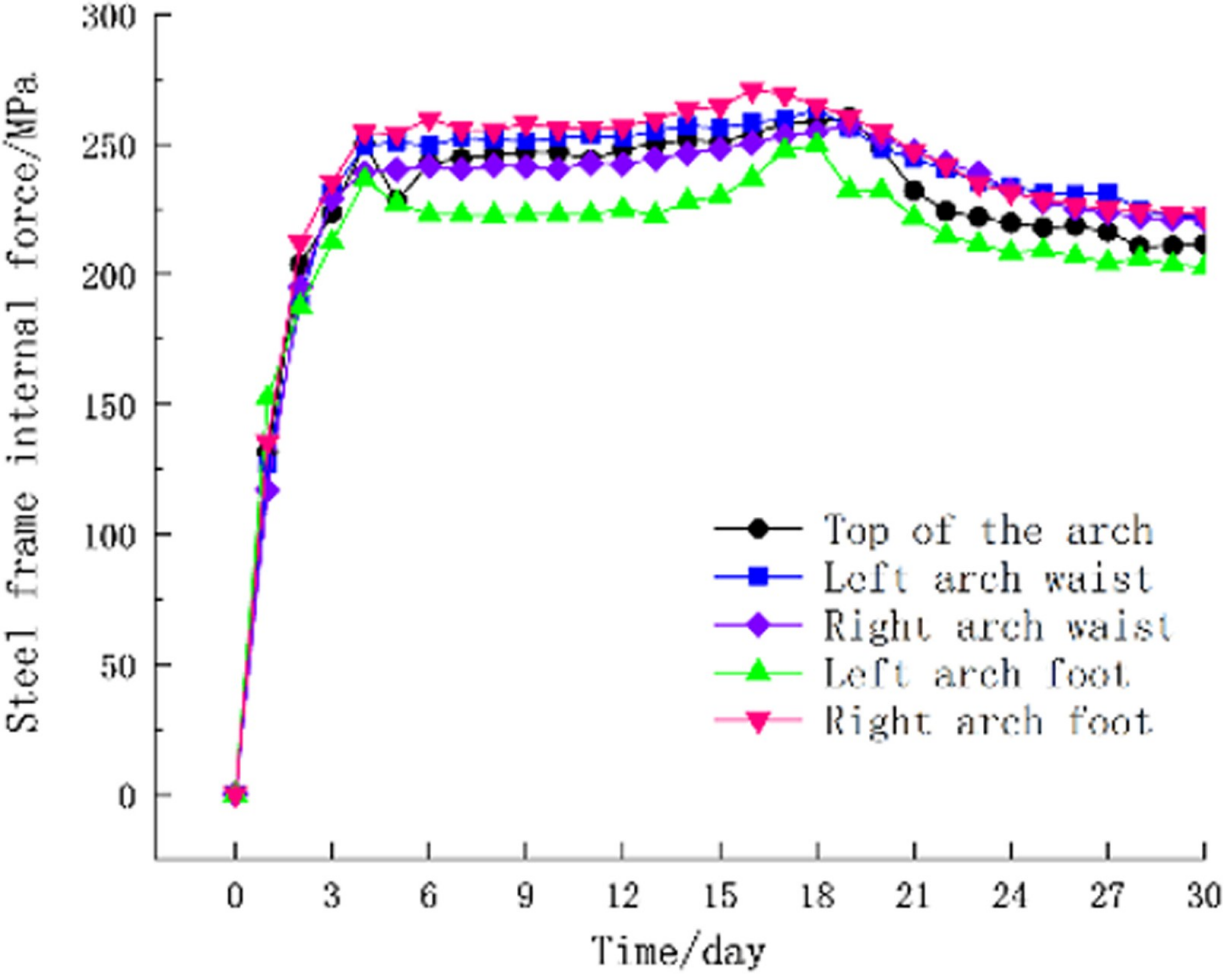
Time-history curve of internal force of steel frame at monitoring section.

**Fig 14:**
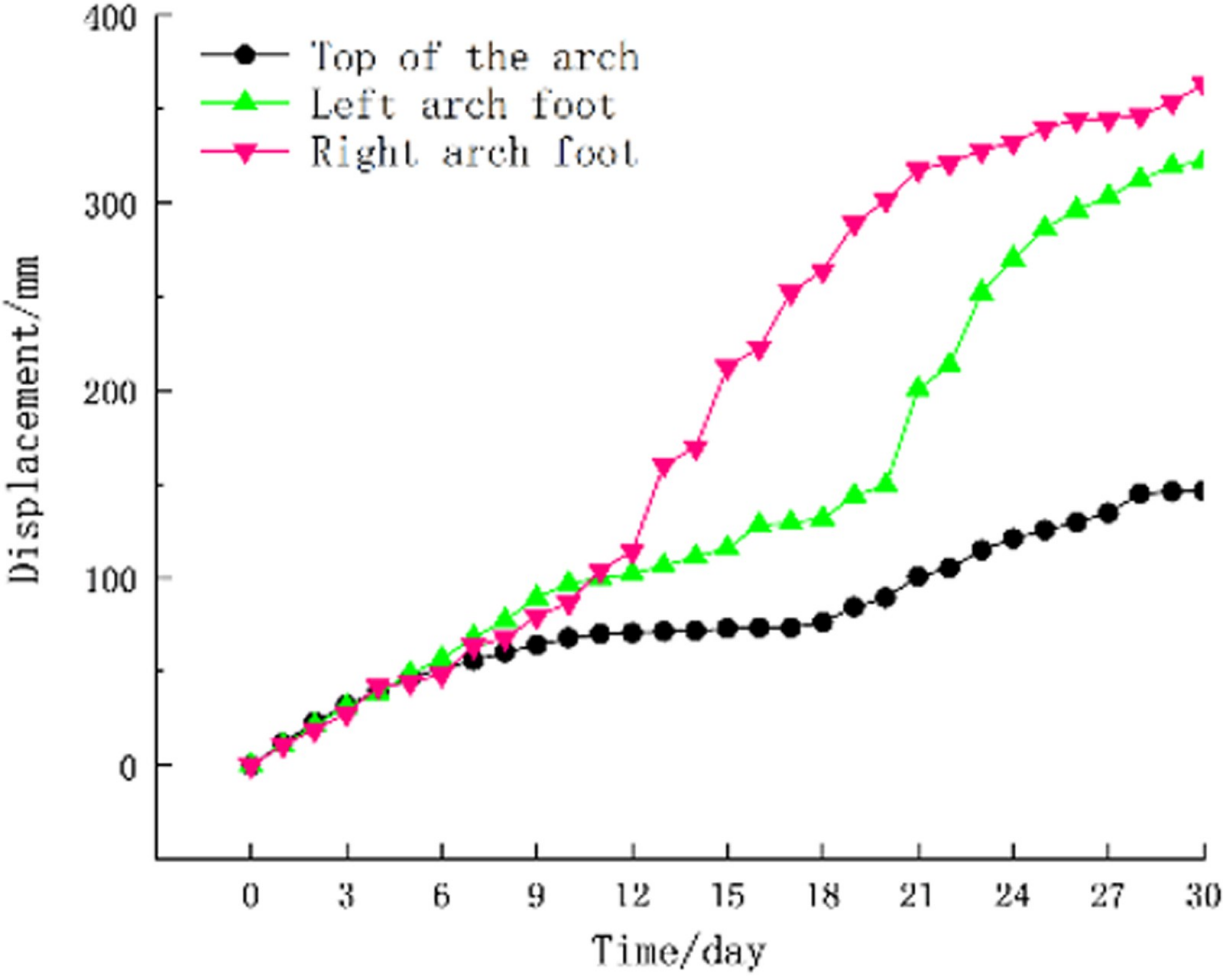
Time-history curve of support convergence displacement at monitoring section.

The monitoring data of the DK59+222 section is used as an example for analysis. Since the monitoring data at the DK59+221.4 and DK59+220.8 sections are similar, they will not be expanded here. The details are as follows:

**(i)** The time history curve of the surrounding rock pressure at the monitoring section, as shown in Fig 12, the surrounding rock pressure is positive with the pressure, and the surrounding rock pressure increases rapidly in the early stage of support. pressure-relief joint begins to show buckling deformation. With the increase of web deformation, the surrounding rock pressure decreases, and the position where the surrounding rock pressure decreases the most is at the right arch.
**(ii)** The time-history curve of the internal force of the steel frame at the monitoring section (Fig 13), the internal force of the steel frame is positive under pressure-relief. From Fig 13, it can be seen that the position of the maximum compressive stress in the steel frame is at the right arch foot, which is the same as the position with the maximum pressure of the surrounding rock. The internal force of all members in the steel frame decreases to varying degrees with the increase of the buckling deformation of the web of the pressure-relief joint, which indicates that the pressure of the surrounding rock is released and the internal force of the steel frame is reduced after the pressure-relief joint is deformed.
**(iii)** The time-history curve of the support convergence displacement in the monitoring section is shown in Fig 14. In the early stage of the support, the deformation rates of the vault and the left and right arch feet are the same. Now, the buckling deformation of the pressure-relief joint web has not occurred. The web begins to deform, the displacement at the right arch is the fastest, and the final deformation is the largest. The final settlement displacement of the vault and the final horizontal convergence displacement of the periphery do not exceed the reserved deformation amount for construction.

**Fig 15:**
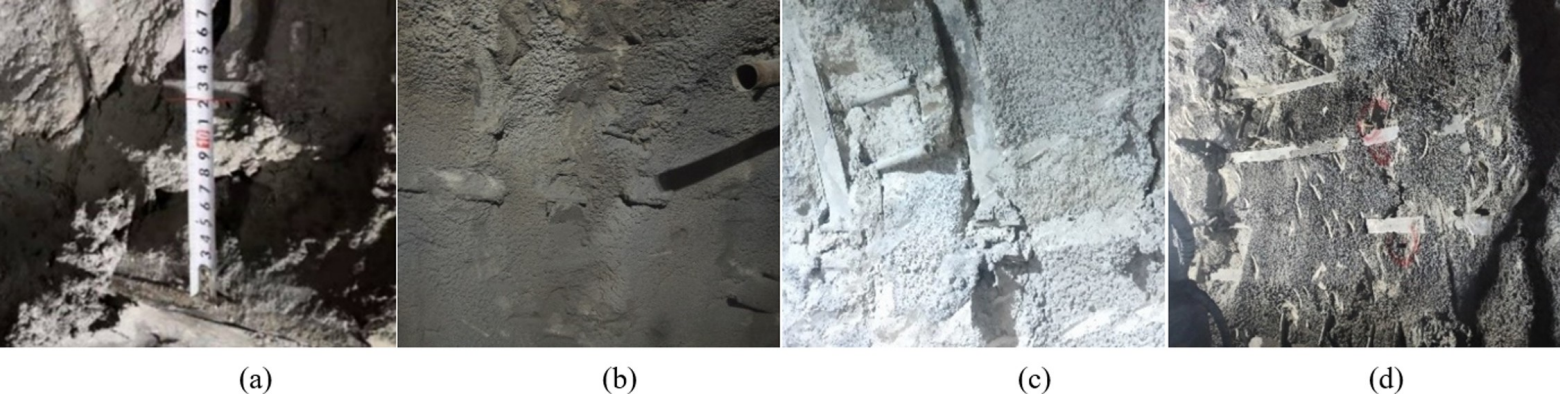
Deformation status of new type yielding support. (a) Pressure-relief joint deform the graph, (b)Left arch pressure-relief joint, (c)Right arch pressure-relief joint, (d)Damage to the top of the arch.

The deformation of the pressure-relief joints in the test arch is shown in Fig 15(A). It can be seen from the picture that after the tunnel is excavated, the supporting structure is mainly subjected to the hoop pressure (the pressure-relief joints are deformed vertically, and there is no apparent lateral deformation). The pressure-relief joint is mainly subjected to axial pressure, and the actual deformation path of the pressure-relief joint is the same as the designed deformation path. Currently, the net height of the pressure-relief joint is 124mm, compressed by 61mm. It can be seen from the monitoring data that the pressure-relief joint is still. There is a pressure-relief margin.

### 5.4 Method comparison

In this study, the maximum internal encroachment displacement of the support structure in a stable state was used as the evaluation parameter. By comparing the numerical simulation results, laboratory experiment results, and field experiment mechanical performances of the pressure-relief joints, the effectiveness and rationality of the pressure-relief joint design were verified.

In the Haba Snow Mountain Tunnel, the design only allows for a maximum horizontal displacement of the left and right arch frames and a maximum subsidence at the arch crown of 500mm. As shown in Fig 8, the laboratory experiment measured a maximum displacement of a single pressure-relief joint at 150mm, with numerical simulation yielding 160mm. Taking the displacement at the right arch foot in Fig 14 as an example, which was 365mm (due to complex geological conditions, this displacement is significantly larger compared to the numerical simulation and laboratory experiment results), it is evident that the maximum displacement values obtained by all three methods are less than the tunnel design’s allowable displacement values, ensuring the safety of the support structure and the rationality of the pressure-relief joint design.

### 5.5 Comparison of support schemes

From the aspects of safety and practicability, comparing the new pressure-relief support scheme proposed in this paper with the original support scheme, it can be seen that the original initial support structure converges and deforms more on the right side of the DK59+225~+300 line. The maximum cumulative horizontal convergence on one side at DK59+240 is 722mm, within the range from the vault to the right arch waist, the concrete is intermittently cracked by about 60m, and a small amount of concrete falls off from the vault to the left arch waist. The new type of pressure-relief support structure is located in the DK59+220.8~+222 section. A small amount of concrete falls off at the pressure-relief joint of the vault and the left arch waist, and much concrete falls off at the pressure-relief joint of the right arch waist. It can be seen that the pressure-relief supports. The safety of the support structure is better than that of the rigid support structure, and the failure phenomenon of the new yielding support structure is shown in Fig 15.

The new pressure-relief support scheme is improved based on the initial support scheme. Only at the connection between the vault of the 25b steel frame and the left and right arch waists is a new type of pressure-relief joint installed, and other procedures are still by the original initial support.

## 6 Discussions and conclusion

This paper focuses on the problem of damage to initial support structures in soft rock tunnels under high ground stress due to large compressive deformations and proposes a new type of pressure-relief joint. Through numerical simulations, laboratory experiments, and field tests on these joints, the following conclusions were drawn:

**(i)** The bearing performance of the pressure-relief joints was studied through numerical simulations and indoor experiments. The results show that the pressure-relief joints, while undergoing significant compressive deformation, still maintain a constant bearing capacity.
**(ii)** According to the monitoring data from the experimental section, the stress in the surrounding rock and steel frames at the right arch foot was high, with significant horizontal convergent displacement. After the pressure relief, the stress in the surrounding rock and steel frames was significantly reduced, demonstrating that the pressure-relief joints achieved the goal of releasing the deformation pressure of the surrounding rock and reducing the internal force in the steel frames through the bending deformation of the web plate.
**(iii)** The field experiments showed that the actual deformation path of the pressure-relief joints matched the preset path. When the support structure and surrounding rock reached a stable state, the initial support structure with newly installed pressure-relief joints did not suffer any damage, indicating that the new type of pressure-relief support joint significantly improves the mechanical performance of the tunnel’s initial support structure.
**(iv)** The pressure-relief joint structure proposed in this paper is overall well-integrated, easy to source materials for, convenient to process, cost-effective, and simple and straightforward to construct, making it highly practical for engineering applications. This provides a reference for the design of initial support structures in soft rock tunnels under high ground stress.
**(v)** However, this study has limitations. The new type of pressure-relief joint only allows for a vertical deformation of 150mm and a horizontal deformation of 50mm, which limits the extent of deformability. Future research should focus on the structural optimization of the joints to enhance the deformability of the pressure-relief joints.

## Supporting information

S1 Data Supplement data about the drawings in this manuscript. (XLSX)
